# Magnetic field-assisted assembly of iron oxide mesocrystals: a matter of nanoparticle shape and magnetic anisotropy

**DOI:** 10.3762/bjnano.10.90

**Published:** 2019-04-17

**Authors:** Julian J Brunner, Marina Krumova, Helmut Cölfen, Elena V Sturm (née Rosseeva)

**Affiliations:** 1 University of Konstanz, Konstanz, Germany

**Keywords:** directed assembly, magnetite, mesocrystal, nanoparticle, transmission electron microscopy

## Abstract

This letter describes the formation and detailed characterization of iron oxide mesocrystals produced by the directed assembly of superparamagnetic iron oxide-truncated nanocubes using the slow evaporation of the solvent within an externally applied homogeneous magnetic field. Anisotropic mesocrystals with an elongation along the direction of the magnetic field can be produced. The structure of the directed mesocrystals is compared to self-assembled mesocrystalline films, which are formed without the influence of a magnetic field. The remarkable structural difference of mesocrystals produced within the external magnetic field from those self-assembled without field indicates that the specific nanoparticle ordering within the superstructure is driven by competing of two types of anisotropic interactions caused by particle shape (i.e., faceting) and orientation of the magnetic moment (i.e., easy axes: <111>_magnetite_). Hence, these findings provide a fundamental understanding of formation mechanisms and structuring of mesocrystals built up from superparamagnetic nanoparticles and how a magnetic field can be used to design anisotropic mesocrystals with different structures.

## Findings

In materials science, nanoparticles and their assemblies belong to the hot research topics nowadays [1–7]. One reason is that their properties can strongly differ from the physical and chemical properties of their corresponding bulk material [8–10]. As a matter of fact, magnetite bulk single crystals are ferrimagnetic, while magnetite nanocrystals with sizes less than 30 nm become superparamagnetic [11–13]. Hence, assemblies consisting of superparamagnetic magnetite nanocrystals are still superparamagnetic although their sizes can be tens or hundreds of micrometres [14–15].

This can be useful to obtain increased magnetization while retaining the superparamagnetic properties of magnetite nanocrystals [16]. Additionally, emergent properties from the single nanocrystals can arise within ordered assemblies, which makes the research on such assemblies highly interesting [16–18].

These ordered assemblies can be formed via different approaches, whereby the self-assembly by evaporation of the solvent of a nanoparticle dispersion is the most prominent one [13,19]. Magnetite nanoparticles can be self-assembled to mesocrystals (i.e., long-range translational and preferable orientational order of nano-sized building blocks) [13–14,20]. Due to the superparamagnetic property of the magnetite nanocrystals, their assembly process can be strongly influenced by an external magnetic field which is then labelled as “directed assembly” [21]. The magnetic moment of the magnetic nanocrystals tends to align along a certain crystallographic axis within a magnetic field, the so-called “easy axis”. Therefore, superparamagnetic magnetite nanocrystals with an easy axis along <111>_magnetite_ give an extraordinary opportunity to investigate the assembly processes directed by an external magnetic field [22–24]. This approach provides an opportunity to further design novel superstructures including mesocrystals with outstanding morphologies and orientational relations of nanocrystals, which cannot be formed otherwise.

In our previous study, the iron oxide nanocrystal synthesis and characterization as well as the formation of self-assembled mesocrystalline mono- and multilayers were presented [13]. The synthesised 10 nm sized superparamagnetic iron oxide nanocrystals have magnetite as the dominant phase and the morphology of a cube truncated by {110}, {111}, {310}, and {114} faces. The morphology of the nanocubes is crucial to further understand the symmetry of packing arrangement and orientational order in 2D and 3D superlattices. The 3D structures of the self-assembled nanocubes can be approximated by a slightly distorted *fcc* superlattice, thereby the texture-like wide-angle diffraction pattern indicates the formation of mesocrystals with a preferred orientational order of nanoparticles. The orientational order of nanoparticles within the superlattice (SL) can be described as follows: [001]_SL_ || [310]_magnetite_, [001]_SL_ || [301]_magnetite_, [001]_SL_ || [100]_magnetite_ [13].

This study is a continuation of our research on iron oxide mesocrystals and focused on the formation and structural characterisation of “directed mesocrystals”. This research demonstrates how the external magnetic field affects the assembly process of magnetite nanocrystals and morphological and structural features of mesocrystals. Furthermore, the alignment of the nanoparticles within the superlattices remarkably differentiates from that obtained without magnetic field.

**Figure 1:**
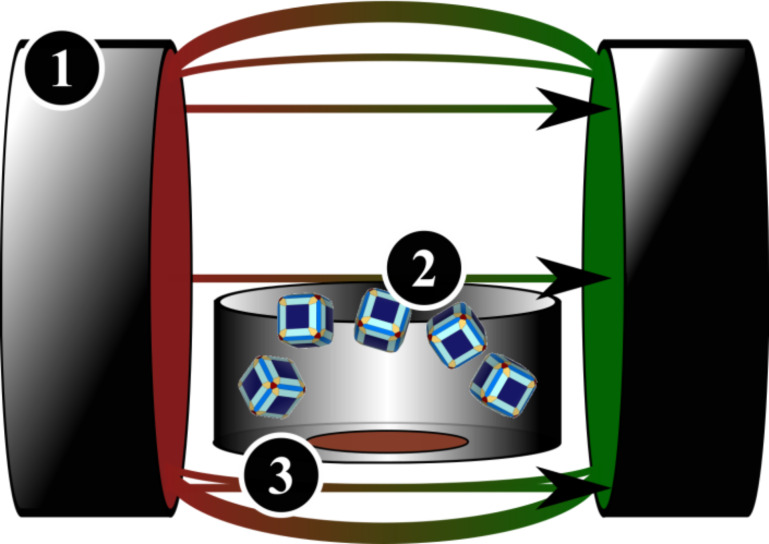
Schematic illustration of the experimental set-up to produce directed mesocrystals. A nanocrystal dispersion (2) was left to dry on top of a TEM grid (3) within an applied homogeneous magnetic field (1). The arrows indicate the direction of an applied external magnetic field.

For the formation of “directed mesocrystals”, a nanocrystal dispersion was left to dry within a homogeneous magnetic field (Figure 1). The homogenous magnetic field with a field strength is 120 mT was created by an electromagnet. The formed superstructures were investigated by collecting the substrate (i.e., TEM grid) after the drying was completed. The substrate was placed on the bottom of the glass vial and was further investigated by methods of scanning and transmission electron microscopy (SEM and TEM).

**Figure 2:**
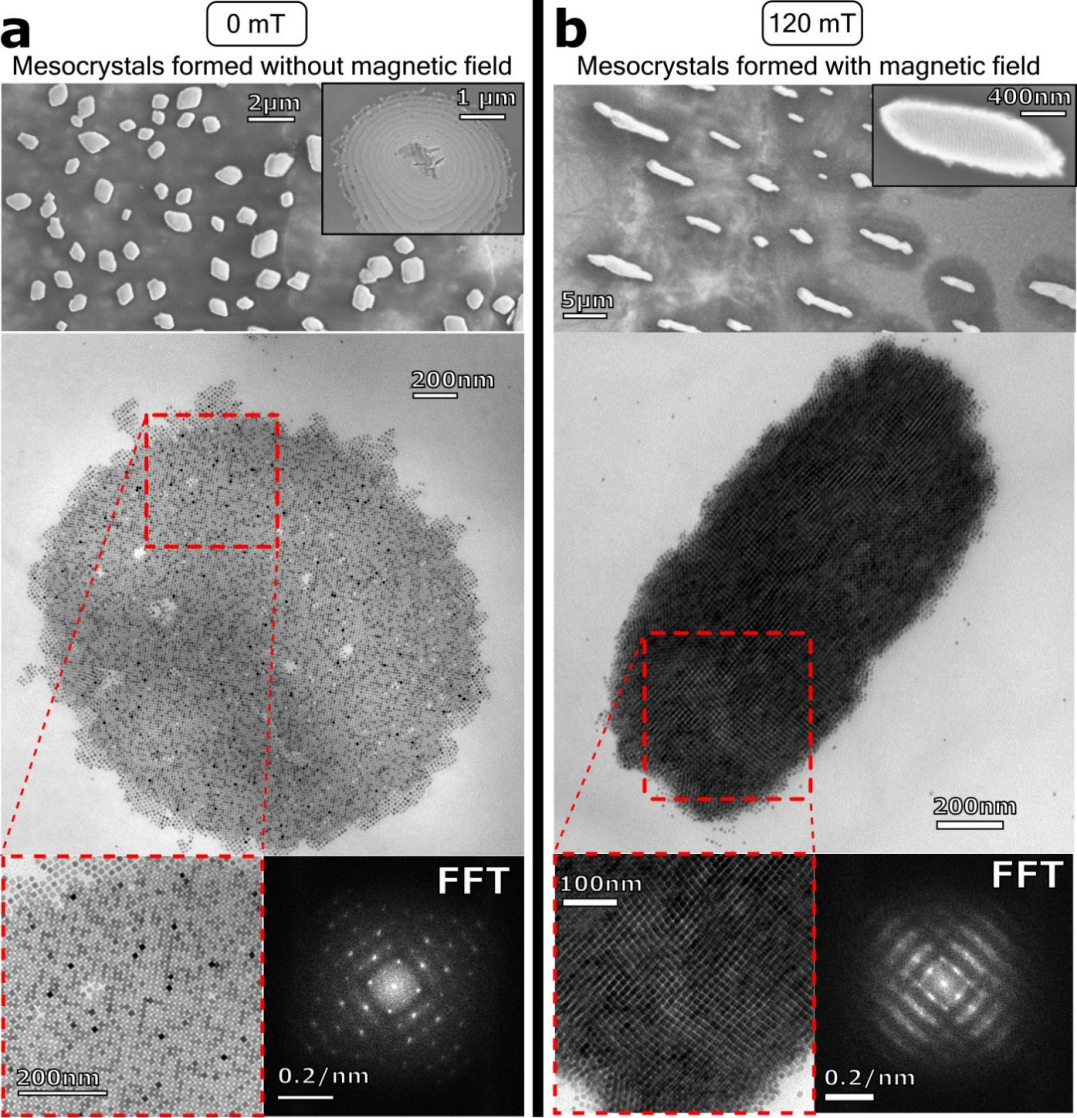
SEM and TEM images of mesocrystals produced without (a) and with (b) external magnetic field. The morphology of the mesocrystals changes using an applied external magnetic field (120 mT). The mesocrystals were elongated in the same direction and aligned along the direction of the used magnetic field.

After the drying process was completed, SEM and TEM investigations of the substrate revealed elongated superstructures, which strongly differ from the superstructures formed without the influence of a magnetic field (Figure 2). Instead of regular mesocrystals, elongated colloidal solids formed. These elongated superstructures with sizes up to several tens of micrometres aligned along the direction of the applied magnetic field (Figure 2b). This observation proves that the assembly process of the nanocrystals to superlattices was significantly affected by an external magnetic field and it further provides opportunities in controlled materials design.

**Figure 3:**
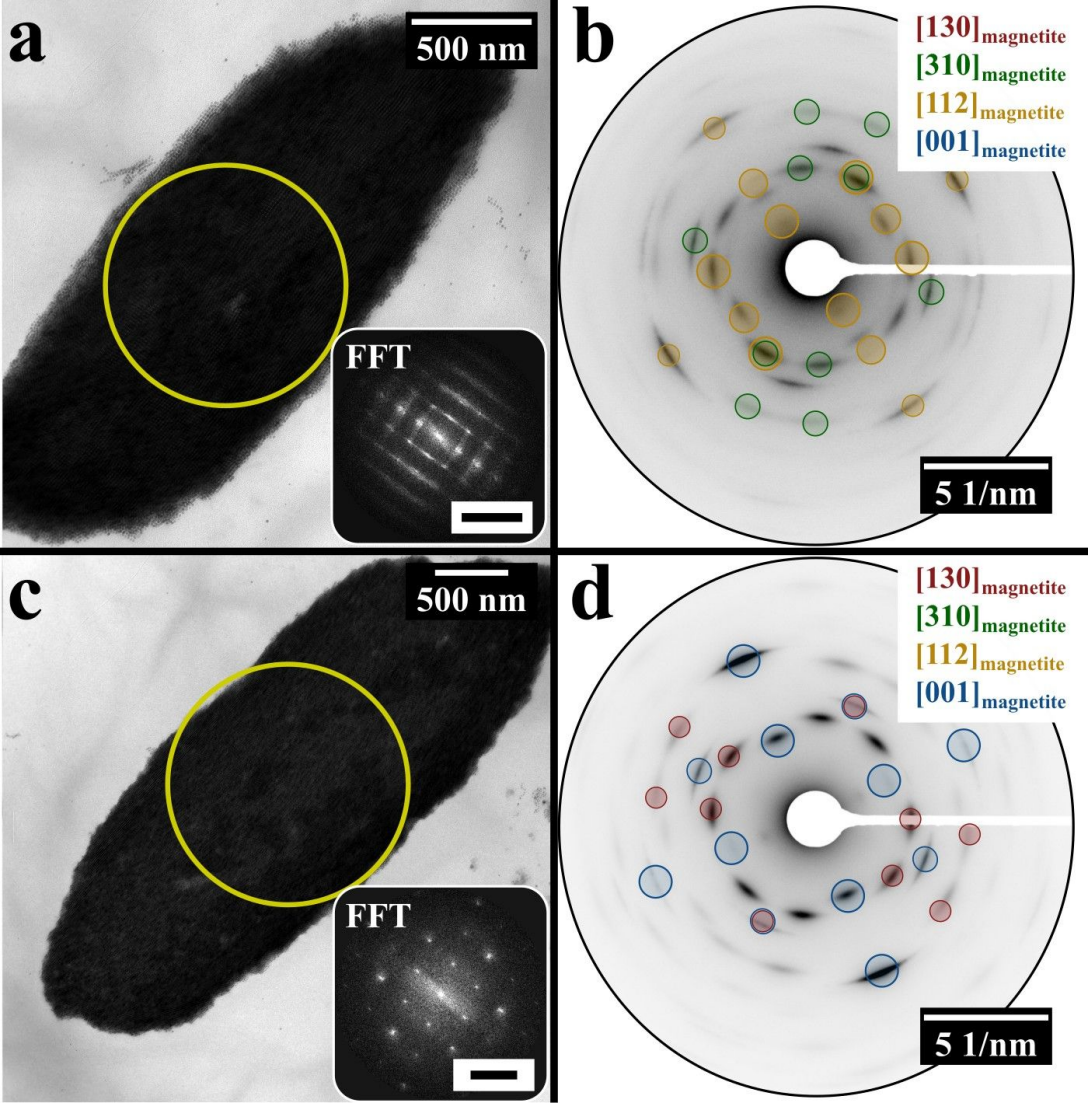
TEM images of two directed mesocrystals and their corresponding ED and FFT patterns. The large yellow circles indicate the positions where the EDs and FFTs were taken. The zone axes contributing to the ED patterns are noted and some diffraction reflections are highlighted and colour coded. (a) A directed mesocrystal with a projected *p*4*mm* layer symmetry (inset: 200 1/µm). (b) The ED pattern of the directed mesocrystal shown in (a). The texture-like diffraction pattern proves the mesocrystalline character of the directed assembly. (c) A directed mesocrystal with a projected *c*2*mm* layer symmetry (inset: 100 1/µm). (d) The ED pattern of the directed mesocrystal shown in (c). The texture-like diffraction pattern slightly differs from the pattern in (b).

In order to obtain structural insights, further TEM and electron diffraction (ED) measurements were performed on selected directed superstructures with sizes in between 2–4 µm. The selected TEM images of the directed superstructures together with their single crystal-like fast Fourier transforms (FFTs) and texture-like EDs are shown in Figure 3. The large yellow circles indicate the position from which the FFTs and EDs are obtained. The symmetry of the projected mesocrystal surface, which is parallel to the substrate can be described either in *p*4*mm* (Figure 3a, inset) or *c*2*mm* (Figure 3c, inset) plane groups. The ED patterns (Figure 3b,d) indicate the preferred crystallographic orientation of the nanocrystals within the superlattice, which is necessary to classify the directed colloidal crystals as mesocrystals (type I) [13,19]. However, the orientational order of the nanocrystals can differ for different mesocrystals.

**Figure 4:**
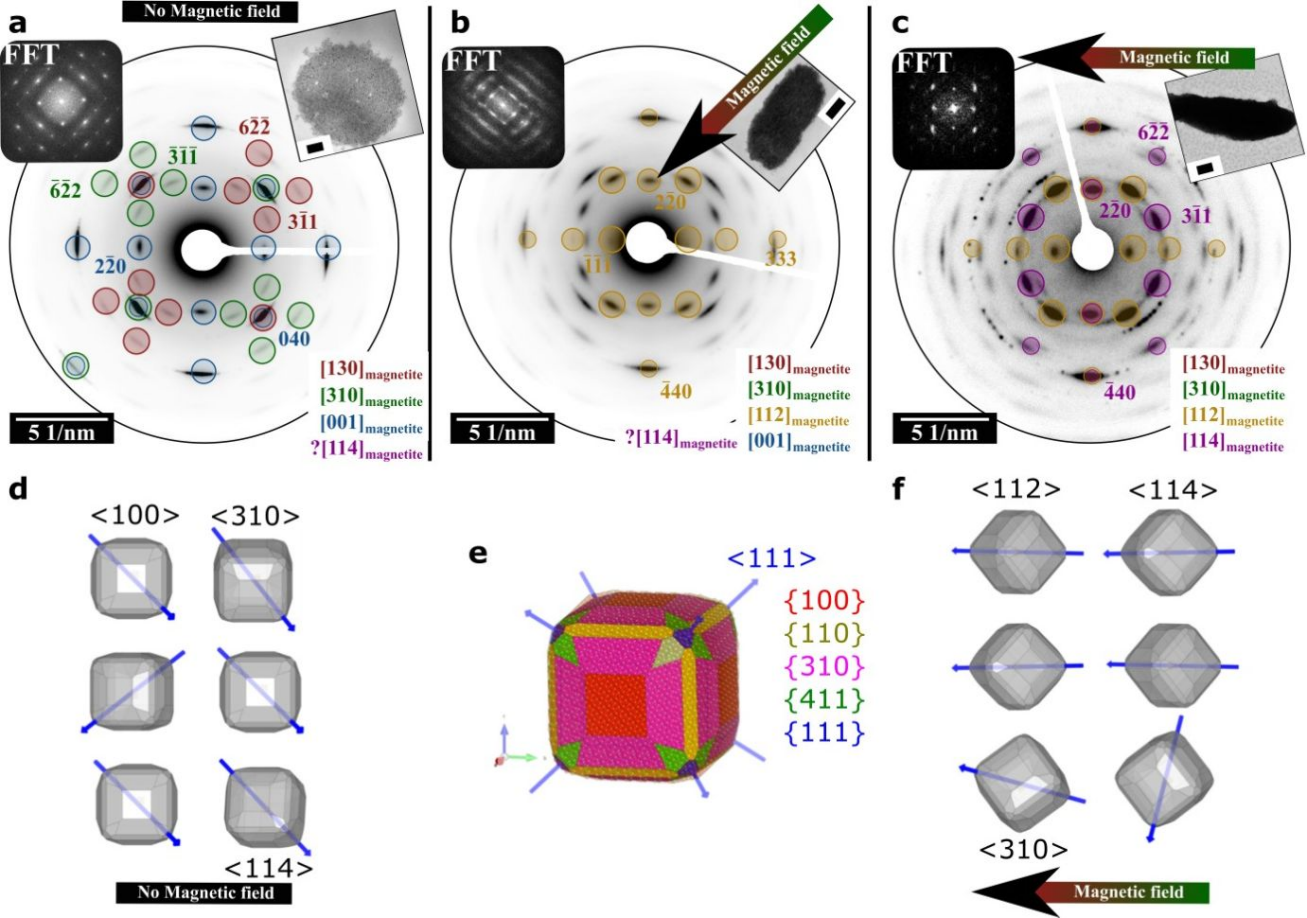
Comparison of a “self-assembled mesocrystal” and two “directed mesocrystals” with a projected *p*4*mm* layer symmetry of superlattices. (a) Indexed SAED pattern of a “self-assembled” mesocrystal (inset: scale bar = 300 nm) formed without the influence of an applied external field. (b,c) Indexed SAED patterns of a “directed mesocrystal” formed within the applied external magnetic field (120 mT). Here, two additional zone axes ([112]_magnetite_ and [114]_magnetite_ perpendicular to the substrate) contribute to the mesocrystalline structure while in (c) the reflections of the [001]_magnetite_ zone axis almost vanish. The diffraction reflections, which are corresponding to the [112]_magnetite_ and [114]_magnetite_ zone axes of magnetite, are indicated in yellow and magenta colour, respectively (inset in (b) and (c): scale bar = 300 nm and 200 nm, respectively). Parts (d) and (f) show illustrations of orientational order of nanoparticles within a projected layer (with *p*4*mm* symmetry) of superlattices prepared without and with magnetic field, respectively. For simplicity only one of all equivalent <111>_magnetite_ directions (corresponding to magnetite easy axis) is indicated (blue arrow). (e) Simulated idealized shape of magnetite nanocube slightly truncated by the {111}, {110}, {310}, and {411} faces. Blue arrows indicate <111>_magnetite_ directions which are corresponding to magnetite easy axis.

The ED patterns obtained for different mesocrystals are visualized in Figure 4 and show the transformation from “self-assembled mesocrystals” to “directed mesocrystals”. For comparison, three structurally different mesocrystals with their corresponding EDs and FFTs are presented. A “self-assembled mesocrystal” is shown next to two “directed mesocrystals”, whereby all superlattices show a projected *p*4*mm* symmetry of the basal surface, which is parallel to the substrate. Nevertheless, the orientational order of magnetite nanocrystals remarkably differs for different mesocrystals. The structure of the “self-assembled” mesocrystalline film produced without external field (Figure 4a,d) was described in great detail in our previous publication [13]. These mesocrystals with an fcc superlattice show the following orientational relationship of magnetite nanocrystals: [001]_SL_ || [310]_magnetite_, [001]_SL_ || [301]_magnetite_, [001]_SL_ || [100]_magnetite_ (the presence of [114]_magnetite_ orientation cannot be excluded since most reflections are overlapped with [310]_magnetite_ and [301]_magnetite_). Thus, it was highlighted that the morphology of the magnetite nanoparticles (Figure 4e) plays a dominant role and controls the symmetry of packing arrangement and the orientational order of the nanoparticles within the mesocrystals. In contrast to such mesocrystals, the selected area electron diffraction (SAED) patterns of the “directed mesocrystals” (Figure 4b,c,f) indicate the presence of the additional nanocrystal orientations within the mesocrystalline structure (aligned perpendicular to the substrate). The additional diffraction reflections can be indexed as [112]_magnetite_ and [114]_magnetite_ zone axis, indicating that some particles within adjacent layers orient with <112>_magnetite_ and <114>_magnetite_ directions perpendicular to the substrate. Moreover, for some “directed mesocrystals” (Figure 3c,d and Figure 4c) the <111>_magnetite_ (lying within the projected plane) is parallel to the substrate and the direction of magnetic field lines. This indicates that the orientation of some nanoparticles within the superlattice with <112>_magnetite_ perpendicular to the substrate might be caused by the simultaneous competition of two types of anisotropic interactions between nanoparticles during the assembly process [24]. On one side the particle shape anisotropy promotes face-to-face alignment (i.e., interaction of the particle {110} faces with other particle faces within the layer) and on the other side magnetic anisotropy of magnetite nanoparticles induces the alignment of the <111>_magnetite_ easy axis parallel to the direction of the external magnetic field. Nevertheless, for some mesocrystals, misalignment of the magnetic easy axis with respect to the direction of the magnetic field was observed (Figure 3a,b and Figure 4b).

Unfortunately, the available data cannot provide the complete description of the 3D structures of the mesocrystals, since the symmetry of the superlattices is unknown. However, based on the results of the wide-angle ED patterns and FFTs of the superlattices obtained from the same position of the selected mesocrystals, the orientations of nanoparticles relative to the crystallographic direction of the superlattice running perpendicular to the imaged plane can be retrieved. In case of mesocrystals shown in Figure 3a and Figure 4, this direction can be indexed as [001]_SL_ (since [001] is perpendicular to the face with *p*4*mm* plane symmetry in both cubic and tetragonal systems). For mesocrystals shown in Figure 3a and Figure 4b, the orientational relations are: [001]_SL_ || [001]_magnetite_, [001]_SL_ || [130]_magnetite_, [001]_SL_ || [310]_magnetite_, [001]_SL_ || [112]_magnetite_, while for the mesocrystal in Figure 4c is: [001]_SL_ || [130]_magnetite_, [001]_SL_ || [310]_magnetite_, [001]_SL_ || [112]_magnetite_, [001]_SL_ || [114]_magnetite_.

In total, 25 of such “directed mesocrystals” were analysed and have shown the same structural variation as described above. These structural features of “directed mesocrystals” indicate that complex interactions between anisotropically shaped superparamagnetic nanoparticles occur during the assembly processes under an external magnetic field. The formation of mesocrystals by solvent evaporation reduces the space of the dispersion volume at the beginning and the mesocrystals form by face-to-face interaction at the end. However, in addition, the magnetic field affects the superparamagnetic nanocrystals. The magnetic field induces the alignment of the magnetic dipole between the nanocrystals and the nanocrystals will then tend to orient their easy axis along the direction of the magnetic field. The magnetic dipole of each nanocrystal will interact with those of the surrounding nanocrystals and they attract to each other with specific crystallographic orientation. Furthermore, during the assembly process, the magnetic field around the nanocrystals might be changed. That, in addition to the particle shape, can be the reason of the misalignment of the <111>_magnetite_ easy axis of magnetite in relation to the mesocrystal elongation and the direction of the external magnetic field. It is also expected that “directed mesocrystals” formed under external magnetic field should demonstrate enhanced anisotropic magnetic properties compared with mesocrystals assembled from the same nanoparticles but without the applied magnetic field.

In summary, we presented a way to form “directed mesocrystals” from superparamagnetic iron oxide nanoparticles using a homogeneous magnetic field. These directed mesocrystals are elongated along the magnetic field and their structure differs from regular self-assembled mesocrystals produced under the same conditions but without the external magnetic field. It is shown that the morphology and magnetic properties of the superparamagnetic nanoparticles are crucial for understanding the packing arrangement and the orientational order in superlattices. Furthermore, the magnetic field restricts the diffusion of nanoparticles in the dispersion. This might significantly influence the morphology of the growing mesocrystals. The specific ordering of nanoparticles induced during the assembly process is controlled by competition of two types of anisotropic interactions: the face-to-face alignment is caused by the particle shape (i.e., faceting), while the alignment along the magnetic field lines is driven by the orientation of the magnetic moment of the nanoparticles (i.e., easy axis: <111>_magnetite_). Future work applying varying magnetic field strength will show the structural variety of the mesocrystals, which is possible by defined balancing of spatial and magnetic nanoparticle interactions. The formation of superstructures of superparamagnetic nanoparticles using a magnetic field is, therefore, a highly interesting approach to form tailor-made materials with outstanding anisotropic structures, morphologies and magnetic properties.

## Experimental

The nanocrystals and the self-assembled mesocrystals were synthesised according to literature [13]. The directed mesocrystals were formed by applying a constant homogeneous magnetic field during the assembly of the nanocrystals by solvent evaporation (toluene). The magnetic field was produced using an electromagnet type B-E1085 from Bruker applying a magnetic field of 120 mT. The formed mesocrystals were investigated using a Zeiss crossbeam scanning electron microscope (SEM) and a Zeiss Libra120 transmission electron microscope (TEM). The Zeiss crossbeam is equipped with an SE2 and an Inlens detector. The TEM Zeiss Libra120 is equipped with a LaB_6_ emitter and a Koehler illumination system and was operated at an accelerated voltage of 120 kV.
